# Supplementary material for: A streamlined approach to structure elucidation using in cellulo crystallized recombinant proteins, InCellCryst
Source: Nat Commun. 2024 Feb 24;15:1709. doi: 10.1038/s41467-024-45985-7 (PMC10894269; doi:10.1038/s41467-024-45985-7)
Supplement: Supplementary file 1 — Supplementary Information [file 41467_2024_45985_MOESM1_ESM.pdf]

# InCellCryst - A streamlined approach to structure elucidation using *in cellulo* crystallized recombinant proteins

## SUPPLEMENTARY INFORMATION

Robert Schönherr[1#], Juliane Boger[1#], J. Mia Lahey-Rudolph[1,2,8#], Mareike Harms[1], Jacqueline Kaiser[1], Sophie Nachtschatt[1], Marla Wobbe[1], Rainer Duden[3], Peter König[4,5], Gleb Bourenkov[6], Thomas R. Schneider[6] and Lars Redecke[1,7*]

**Supplementary Figure 1**

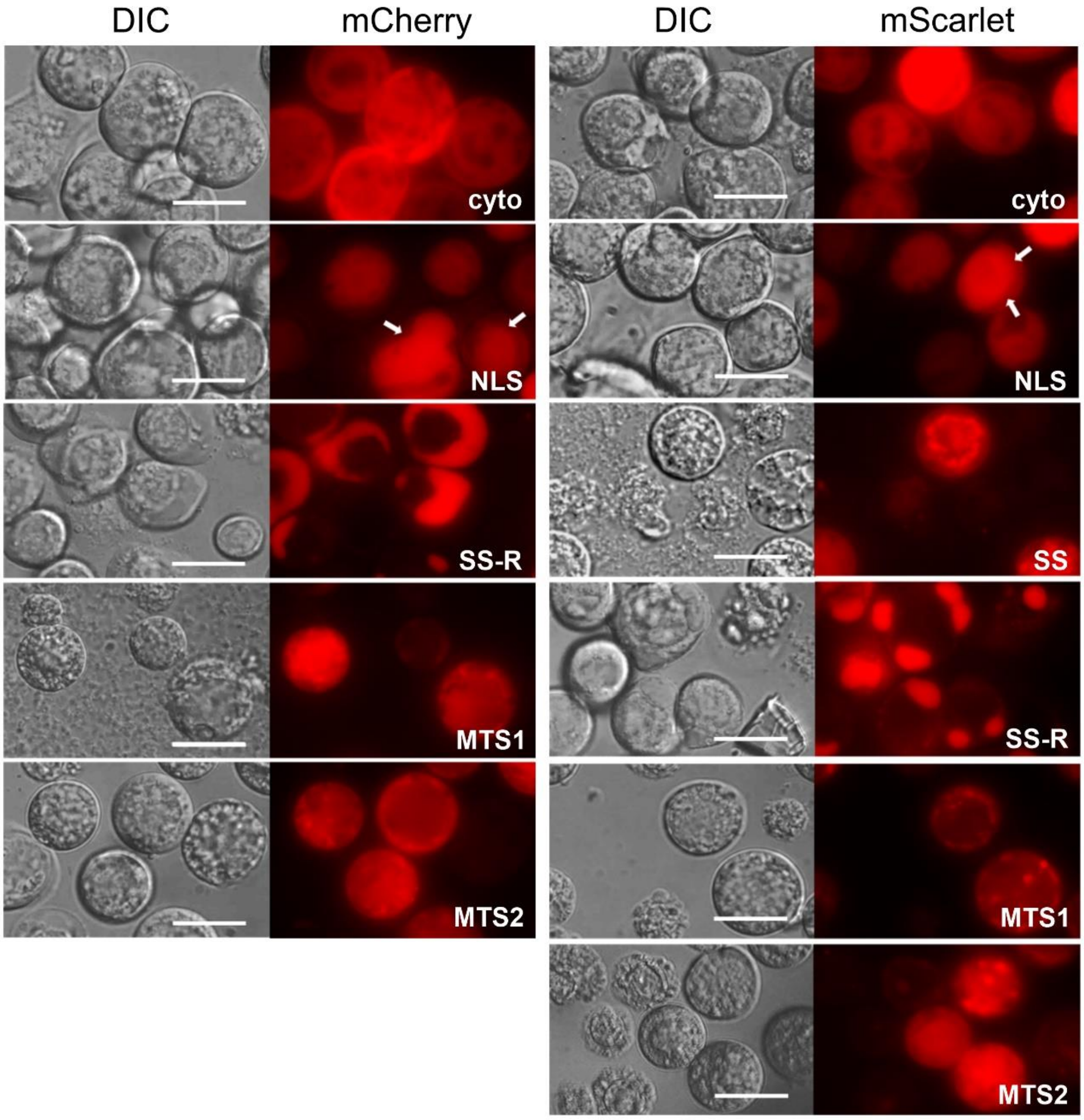


**Supplementary Figure 1: Compartment screening of mCherry-µNS and mScarlett-I-µNS.** rBVs were used to infect High Five insect cells at an MOI of 1. Representative images were taken 5 dpi on a Nikon Ts2R-FL microscope equipped with 100x objectives using the DIC contrast mode and mCherry or mScarlett-I wide-field fluorescence. The rBV encoded cellular translocation signals are indicated: cyto, cytosol; NLS, nuclear localization sequence; SS, N-terminal signal sequence for ER import; SS-R, N-terminal signal sequence for ER import and C-terminal ER retention signal; MTS1, mitochondrial localization sequence 1; MTS2, mitochondrial localization sequence 2. Size bars for all images represent 20 µm. Indications for intracellular crystallization were not observed. Similar results have been obtained in three biologically independent experiments.

**Supplementary Figure 2**

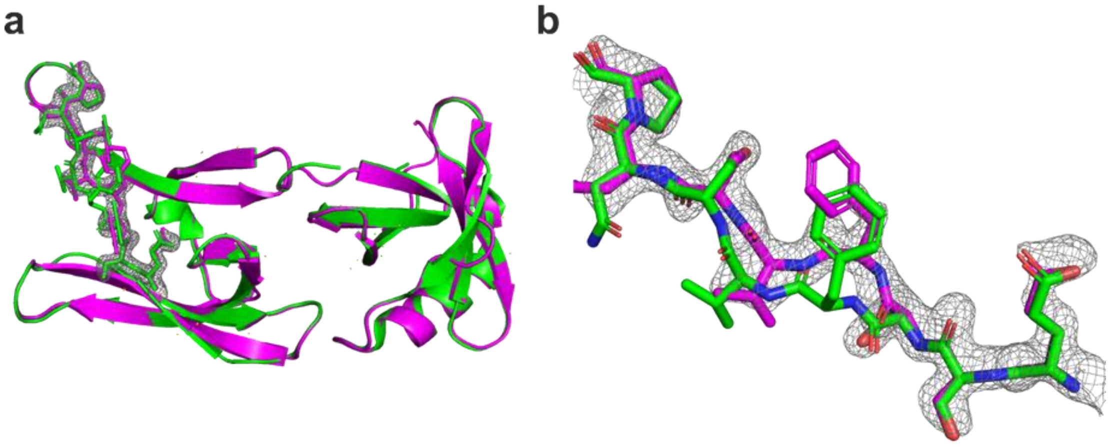


**Supplementary Figure 2: Omit maps of a representative region of the HEX-1 ori structure. a**, Overlay of HEX-1 crystallized in a sitting drop (PDB 1KHI; shown in green) with the *in cellulo* crystallized HEX-1 ori (processed using *CrystFEL*; shown in pink). A representative section of the Fo-Fc map is shown as gray mesh at 3.0 sigma. **b**, Enlarged view of the differences between the search model (green) and the HEX-1 ori (*CrystFEL*) structure (violet) solved in this study.

# Supplementary Figure 3

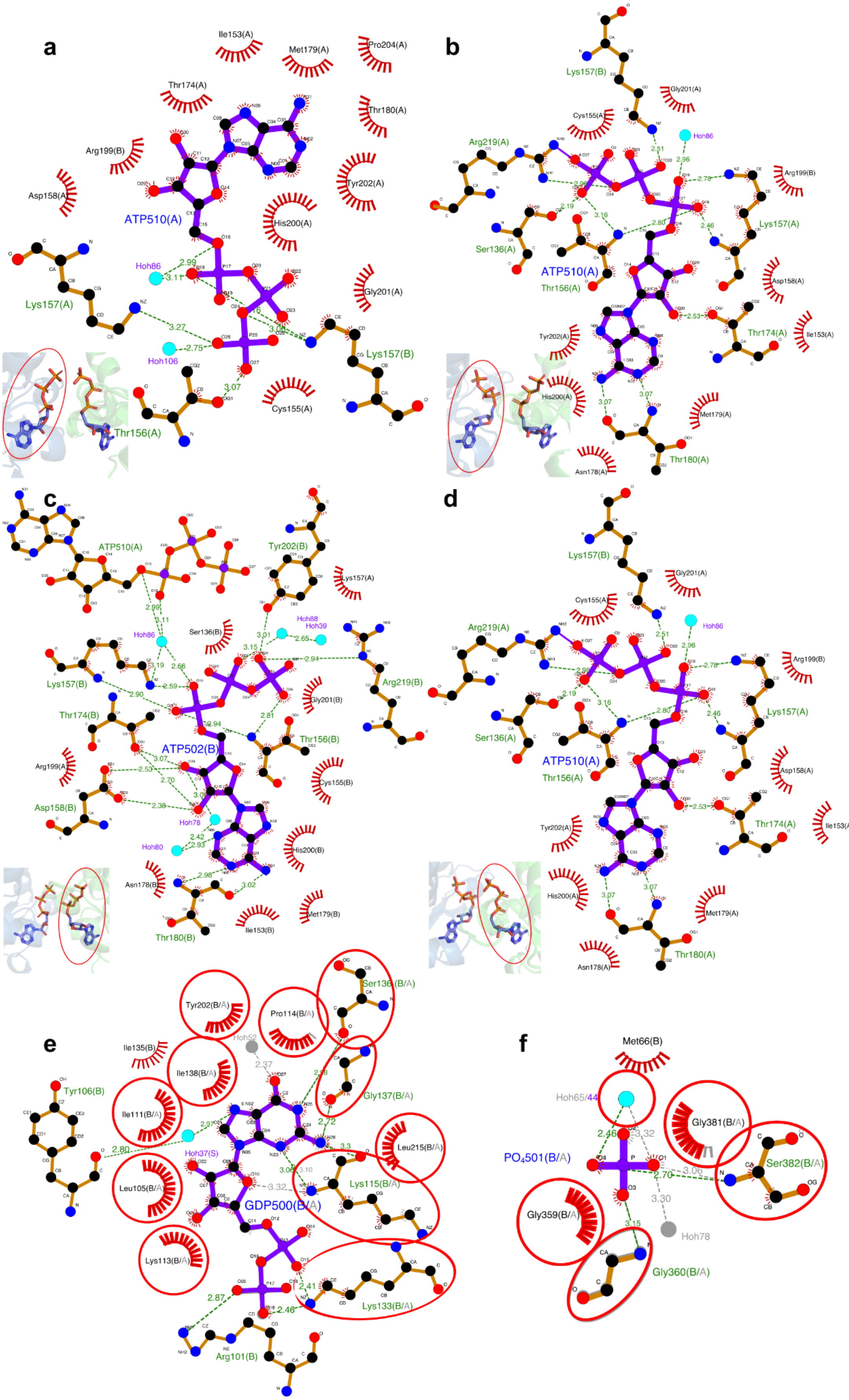

**Supplementary Figure 3: Ligand-protein interactions in the IMPDH ori structure. a-d**, Interactions of ATP molecules in the canonical binding site 1 are depending on the ATP conformation. An Overview of the depicted ATP conformation is shown in every bottom left corner with the detailed ATP molecule encircled in red. Hydrophobic interactions are shown as red half-suns, hydrogen bonds with distances in Å by dashed green lines, solvent water as turquoise dots. **a**, Interactions of ATP with the A-chain in the inward-facing γ-phosphates conformation. **b**, ATP interactions with the A-chain in the outward-facing γ-phosphates conformation. **c**, Interactions of ATP with the B-chain in the conformation of γ-phosphates pointing in the same, right direction. **d**, ATP interactions with the A-chain in the left-oriented gamma phosphates conformation. **e**, GDP bound in the second conserved nucleotide binding site of the bateman domain (canonical binding site 2) is mainly stabilized by hydrophobic interactions. Interactions are equivalent in the IMPDH ori structure solved using *XDS*, which is not shown here, except for the hydrogen bond with Tyr106. This is connected to adenine over a conserved water, only resolved in the higher-resolution *CrystFEL* IMPDH ori model. **f**, Interactions of a phosphate bound to the IMP binding moiety. **e-f**, Interactions of the GDP and phosphate ligands with the A-chain are overlaid to the ligand interactions with the B-chain. Equivalent interacting side chains are encircled in red. All interaction maps were drawn using *LigPlot*$^+$ v.2.2.

**Supplementary Table 1. Overview of all vectors created for the *in cellulo* screening system.** Indicated are the purpose of each vector and the DNA sequence that has been cloned between the HindIII and BamHI sites of the pFastBac1 vector. Denoted are the vector-encoded amino acid sequences that are added to the N- and C-terminus of the target protein. ‘ - protease cleavage site.

| **Cloning system** | **Vector category** | **Vector name** | **Target compartment** | **N-terminal amino acid sequence** | **C-terminal amino acid sequence** | **Base pair sequence between HindIII and BamHI restriction enzyme sites** |
|---|---|---|---|---|---|---|
| **First generation** | Screening | pFB1 cyto | Cytoplasm | MG | A | ATGGGCGCCTAA |
| | | pFB1 PTS1 | Peroxisome | MG | ASKL | CGGTCCGAAGCGCGCGGAATTCATCATGGG CGCCAGCAAACTGTAA |
| | | pFB1 SS | Secretory pathway | MHLMRACITFCIASTAV VAVNA‘G | A | ATGCATCTCATGCGTGCCTGCATCACATTTT GTATCGCTTCGACGGCTGTAGTCGCCGTAAA CGCCGGCGCCTAA |
| | | pFB1 SS-R | Endoplasmic reticulum | MHLMRACITFCIASTAV VAVNA‘G | AKDEL | ATGCATCTCATGCGTGCCTGCATCACATTTT GTATCGCTTCGACGGCTGTAGTCGCCGTAAA CGCCGGCGCCAAAGATGAACTGTAA |
| | Immuno-fluorescence | pFB1 cyto HA-C | Cytoplasm | MG | AYPYDVPDYA | ATGGGCGCCTACCCCTACGACGTGCCCGAC TACGCTTAA |
| | | pFB1 cyto HA-N | Cytoplasm | MYPYDVPDYAG | A | ATGTACCCCTACGACGTGCCCGACTACGCT GGCGCCTAA |
| | | pFB1 SS-R HA-C | Endoplasmic reticulum | MHLMRACITFCIASTAV VAVNAG | AYPYDVPDYAK DEL | ATGCATCTCATGCGTGCCTGCATCACATTTT GTATCGCTTCGACGGCTGTAGTCGCCGTAAA CGCCGGCGCCTACCCCTACGACGTGCCCGA CTACgccAAAGATGAACTGTAA |
| | | pFB1 SS-R HA-N | Endoplasmic reticulum | MHLMRACITFCIASTAV VAVNAYPYDVPDYAG | AKDEL | ATGCATCTCATGCGTGCCTGCATCACATTTT GTATCGCTTCGACGGCTGTAGTCGCCGTAAA |

| | | | | | | |
|---|---|---|---|---|---|---|
| | | | | | | CGCCggcTACCCCTACGACGTGCCCGACTACGCTGGCGCCAAAGATGAACTGTAA |
| | Purification | pFB1 N-His | Cytoplasm | MSYYHHHHHHDYDIPTTENLYFQ‘GAMGSMG | A | ATGTCGTACTACCATCACCATCACCATCACGATTACGATATCCCAACGACCGAAAACCTGTATTTTCAGGGAGCCATGGGATCCATGGGCGCCTAA |
| | | pFB1 N-His HA-C | Cytoplasm | MSYYHHHHHHDYDIPTTENLYFQ‘GAMGSMG | AYPYDVPDYA | ATGTCGTACTACCATCACCATCACCATCACGATTACGATATCCCAACGACCGAAAACCTGTATTTTCAGGGAGCCATGGGATCCATGGGCGCCTACCCCTACGACGTGCCCGACTACGCTTAA |
| **Second generation** | Screening | pFB1 v2 cyto | Cytoplasm | MGT | AS | ATGGGTACCATTGTAGAGGATATTCCCGCCGACAGCTAGCTAA |
| | | pFB1 v2 PTS1 | Peroxisome | MGT | ASKL | ATGGGTACCGAAGCTACTTAGCTGAGATTTGCCCGCTAGCAAGCTGTAA |
| | | pFB1 v2 NLS | Nucleus | MGT | ASPAAKRVKLD | ATGGGTACCCGTCGTCAAGGAACAATCTTTGCTGGCTAGCCCTGCTGCTAAGAGAGTCAAGCTGGACTAA |
| | | pFB1 v2 MTS1 | Mitochondrial matrix | MAARLLLRSLRVLSARSAPRPLPSARC‘SHSGT | AS | ATGGCTGCTAGACTGCTGCTGCGTTCTCTGAGAGTCCTGTCTGCTAGATCCGCTCCTAGACCTCTGCCTTCTGCTAGATGCTCTCACTCTGGTACCTATACGCTGGTGCCAGTCAAATTGCGCTAGCTAA |
| | | pFB1 v2 MTS2 | Mitochondrial matrix | MATAIRLLGRRVSSWRLRPSPSPLAVPRRA‘SHSGT | AS | ATGGCTACCGCCATCAGACTGCTCGGTAGACGTGTCTCTTCCTGGAGACTCAGACCTTCTCCCTCCCCTCTGGCTGTCCCTCGTAGAGCTTCTCACTCTGGTACCACTTCGTCGCATTGTGTAGGCCAAAGCTAGCTAA |
| | | pFB1 v2 SS | Secretory pathway | MHLMRACITFCIASTAVVAVNA‘GT | AS | ATGCACCTGATGAGAGCTTGCATCACCTTCTGCATCGCTTCTACCGCTGTCGTCGCTGTCAA |

| | | | | | | |
|---|---|---|---|---|---|---|
| | | | | | | CGCTGGTACCTCCCCTATGAACGATGTGTCGTGAAGCTAGCTAA |
| | | pFB1 v2 SS-R | Endoplasmic reticulum | MHLMRACITFCIASTAVVAVNA‘GT | ASKDEL | ATGCACCTGATGAGAGCTTGCATCACCTTCTGCATCGCTTCTACCGCTGTCGTCGCTGTCAACGCTGGTACCTTCGATGGCTCTAATTGCAACAGGGGCTAGCAAGGACGAACTGTAA |
| | Immuno-fluorescence | pFB1 v2 cyto HA-N | Cytoplasm | MYPYDVPDYAGT | AS | ATGTACCCTTACGACGTGCCTGACTACGCTGGTACCGTTTCTTACGCAGGCCACGTAAGTAGCTAGCTAA |
| | | pFB1 v2 cyto HA-C | Cytoplasm | MGT | ASYPYDVPDYA | ATGGGTACCTATGCTTGGAGTCCTAACCCAGTAGGCTAGCTACCCTTACGACGTGCCTGACTACGCCTAA |
| | | pFB1 v2 PTS1 HA-C | Peroxisome | MGT | ASYPYDVPDYASKL | ATGGGTACCCAGACTTATGCGTGGTTACGACTCAGCTAGCTACCCTTACGACGTGCCTGACTACGCTTCTAAGCTGTAA |
| | | pFB1 v2 NLS HA-C | Nucleus | MGT | ASYPYDVPDYAPAAKRVKLD | ATGGGTACCCGAGATAGGATCTCGCTTCACGTTAGCTAGCTACCCTTACGACGTGCCTGACTACGCTCCTGCTGCTAAGAGAGTCAAGCTGGACTAA |
| | | pFB1 v2 MTS1 HA-C | Mitochondrial matrix | MAARLLLRSLRVLSARSAPRPLPSARC‘SHSGT | ASYPYDVPDYA | ATGGCTGCTAGACTGCTGCTGCGTTCTCTGAGAGTCCTGTCTGCTAGATCCGCTCCTAGACCTCTGCCTTCTGCTAGATGCTCTCACTCTGGTACCGTAACGTTGGGTTCTCTAAGCGTCCGCTAGCTACCCTTACGACGTGCCTGACTACGCCTAA |
| | | pFB1 v2 MTS2 HA-C | Mitochondrial matrix | MATAIRLLGRRVSSWRLRPSPSPLAVPRRA‘SHSGT | ASYPYDVPDYA | ATGGCTACCGCCATCAGACTGCTCGGTAGACGTGTCTCTTCCTGGAGACTCAGACCTTCTCCCTCCCCTCTGGCTGTCCCTCGTAGAGCTTCTCACTCTGGTACCGCCTCAAGGAGAGTTTGGTCCTAATGCTAGCTACCCTTACGACGTGCCTGACTACGCCTAA |

| | | | | | | |
|---|---|---|---|---|---|---|
| | | pFB1 v2 SS-R HA-C | Endoplasmic reticulum | MKLSLVAAML LLLSAARA‘GT | ASYPYDVPDYAK DEL | ATGCACCTGATGAGAGCTTGCATCACCTTCT GCATCGCTTCTACCGCTGTCGTCGCTGTCAA CGCTGGTACCTATCCGTAAGTTCGTAAGCAC CTGGGCTAGCTACCCTTACGACGTGCCTGA CTACGCTAAGGACGAACTGTAA |
| | | pFB1 v2 SS-R HA-N | Endoplasmic reticulum | MKLSLVAAML LLLSAARA‘YPYDVPDY AGT | ASKDEL | ATGCACCTGATGAGAGCTTGCATCACCTTCT GCATCGCTTCTACCGCTGTCGTCGCTGTCAA CGCTTACCCTTACGACGTGCCTGACTACGCT GGTACCATCGGGAGATTCGCAAACGTTTCTC GCTAGCAAGGACGAACTGTAA |
| | Fluorescence | pFB1 v2 cyto mTurq2-C | Cytoplasm | GT | ASVSKGEELFTG VVPILVELDGDV NGHKFSVSGEG EGDATYGKLTLK FICTTGKLPVPW PTLVTTLSWGVQ CFARYPDHMKQ HDFFKSAMPEG YVQERTIFFKDD GNYKTRAEVKFE GDTLVNRIELKGI DFKEDGNILGHK LEYNYFSDNVYI TADKQKNGIKAN FKIRHNIEDGGV QLADHYQQNTPI GDGPVLLPDNH YLSTQSKLSKDP NEKRDHMVLLEF VTAAGITLGMDE LYK | ATGGGTACCATTGTAGAGGATATTCCCGCCG ACAGCTAGCGTGAGCAAGGGCGAGGAGCTG TTCACCGGGGTGGTGCCCATCCTGGTCGAG CTGGACGGCGACGTAAACGGCCACAAGTTC AGCGTGTCCGGCGAGGGCGAGGGCGATGC CACCTACGGCAAGCTGACCCTGAAGTTCATC TGCACCACCGGCAAGCTGCCCGTGCCCTGG CCCACCCTCGTGACCACCCTGTCCTGGGGC GTGCAGTGCTTCGCCCGCTACCCCGACCAC ATGAAGCAGCACGACTTCTTCAAGTCCGCCA TGCCCGAAGGCTACGTCCAGGAGCGCACCA TCTTCTTCAAGGACGACGGCAACTACAAGAC CCGCGCCGAGGTGAAGTTCGAGGGCGACAC CCTGGTGAACCGCATCGAGCTGAAGGGCAT CGACTTCAAGGAGGACGGCAACATCCTGGG GCACAAGCTGGAGTACAACTACTTTAGCGAC AACGTCTATATCACCGCCGACAAGCAGAAGA ACGGCATCAAGGCCAACTTCAAGATCCGCC ACAACATCGAGGACGGCGGCGTGCAGCTCG CCGACCACTACCAGCAGAACACCCCCATCG GCGACGGCCCCGTGCTGCTGCCCGACAACC ACTACCTGAGCACCCAGTCCAAGCTGAGCA AAGACCCCAACGAGAAGCGCGATCACATGG TCCTGCTGGAGTTCGTGACCGCCGCCGGGA TCACTCTCGGCATGGACGAGCTGTACAAGTA A |

| | | | | | | |
|---|---|---|---|---|---|---|
| | | pFB1 v2 MTS1 mTurq2-C | Mitochondrial matrix | MAARLLLRSLRVLSARSAPRPLPSARC‘SHSGT | ASVSKGEELFTGVVPILVELDGDVNGHKFSVSGEGEGDATYGKLTLKFICTTGKLPVPWPTLVTTLSWGVQCFARYPDHMKQHDFFKSAMPEGYVQERTIFFKDDGNYKTRAEVKFEGDTLVNRIELKGIDFKEDGNILGHKLEYNYFSDNVYITADKQKNGIKANFKIRHNIEDGGVQLADHYQQNTPIGDGPVLLPDNHYLSTQSKLSKDPNEKRDHMVLLEFVTAAGITLGMDELYK | ATGGCTGCTAGACTGCTGCTGCGTTCTCTGAGAGTCCTGTCTGCTAGATCCGCTCCTAGACCTCTGCCTTCTGCTAGATGCTCTCACTCTGGTACCGTAACGTTGGGTTCTCTAAGCGTCCGCTAGCGTGAGCAAGGGCGAGGAGCTGTTCACCGGGGTGGTGCCCATCCTGGTCGAGCTGGACGGCGACGTAAACGGCCACAAGTTCAGCGTGTCCGGCGAGGGCGAGGGCGATGCCACCTACGGCAAGCTGACCCTGAAGTTCATCTGCACCACCGGCAAGCTGCCCGTGCCCTGGCCCACCCTCGTGACCACCCTGTCCTGGGGCGTGCAGTGCTTCGCCCGCTACCCCGACCACATGAAGCAGCACGACTTCTTCAAGTCCGCCATGCCCGAAGGCTACGTCCAGGAGCGCACCATCTTCTTCAAGGACGACGGCAACTACAAGACCCGCGCCGAGGTGAAGTTCGAGGGCGACACCCTGGTGAACCGCATCGAGCTGAAGGGCATCGACTTCAAGGAGGACGGCAACATCCTGGGGCACAAGCTGGAGTACAACTACTTTAGCGACAACGTCTATATCACCGCCGACAAGCAGAAGAACGGCATCAAGGCCAACTTCAAGATCCGCCACAACATCGAGGACGGCGGCGTGCAGCTCGCCGACCACTACCAGCAGAACACCCCCATCGGCGACGGCCCCGTGCTGCTGCCCGACAACCACTACCTGAGCACCCAGTCCAAGCTGAGCAAAGACCCCAACGAGAAGCGCGATCACATGGTCCTGCTGGAGTTCGTGACCGCCGCCGGGATCACTCTCGGCATGGACGAGCTGTACAAGTAA |
| | | pFB1 v2 MTS2 mTurq2-C | Mitochondrial matrix | MATAIRLLGRRVSSWRLRPSPSPLAVPRRA‘SHSGT | ASVSKGEELFTGVVPILVELDGDVNGHKFSVSGEGEGDATYGKLTLKFICTTGKLPVPWPTLVTTLSWGVQCFARYPDHMKQHDFFKSAMPEGYVQERTIFFKDD | ATGGCTACCGCCATCAGACTGCTCGGTAGACGTGTCTCTTCCTGGAGACTCAGACCTTCTCCCTCCCCTCTGGCTGTCCCTCGTAGAGCTTCTCACTCTGGTACCGCCTCAAGGAGAGTTTGGTCCTAATGCTAGCGTGAGCAAGGGCGAGGAGCTGTTCACCGGGGTGGTGCCCATCCTGGTCGAGCTGGACGGCGACGTAAACGGCCACAAGTTCAGCGTGTCCGGCGAGGGCGAGGGCGATGCCACCTACGGCAAGCTGACCCTGAAGTT |

| | | | | | | |
|---|---|---|---|---|---|---|
| | | | | | GNYKTRAEVKFE GDTLVNRIELKGI DFKEDGNILGHK LEYNYFSDNVYI TADKQKNGIKAN FKIRHNIEDGGV QLADHYQQNTPI GDGPVLLPDNH YLSTQSKLSKDP NEKRDHMVLLEF VTAAGITLGMDE LYK | CATCTGCACCACCGGCAAGCTGCCCGTGCC CTGGCCCACCCTCGTGACCACCCTGTCCTG GGGCGTGCAGTGCTTCGCCCGCTACCCCGA CCACATGAAGCAGCACGACTTCTTCAAGTCC GCCATGCCCGAAGGCTACGTCCAGGAGCGC ACCATCTTCTTCAAGGACGACGGCAACTACA AGACCCGCGCCGAGGTGAAGTTCGAGGGC GACACCCTGGTGAACCGCATCGAGCTGAAG GGCATCGACTTCAAGGAGGACGGCAACATC CTGGGGCACAAGCTGGAGTACAACTACTTTA GCGACAACGTCTATATCACCGCCGACAAGC AGAAGAACGGCATCAAGGCCAACTTCAAGAT CCGCCACAACATCGAGGACGGCGGCGTGCA GCTCGCCGACCACTACCAGCAGAACACCCC CATCGGCGACGGCCCCGTGCTGCTGCCCGA CAACCACTACCTGAGCACCCAGTCCAAGCT GAGCAAAGACCCCAACGAGAAGCGCGATCA CATGGTCCTGCTGGAGTTCGTGACCGCCGC CGGGATCACTCTCGGCATGGACGAGCTGTA CAAGTAA |
| | Purification | pFB1 v2 N-His | Cytoplasm | MSYYHHHHHHDYDYP YDVPDYAIPTTENLYFQ ‘SGT | AS | ATGTCTTACTACCACCATCACCACCATCACG ACTACGACTACCCTTACGACGTGCCTGACTA CGCTATCCCTACCACCGAAAACCTGTACTTC CAGTCTGGTACCTTGTCTAAGCCGAACGTAT CGCATGGCTAGCTAA |
| | | pFB1 v2 SS N-His | Secretory pathway | MKLSLVAAML LLLSAARA‘SYYHHHHH HDYDYPYDVPDYAIPT TENLYFQ‘SGT | AS | ATGCACCTGATGAGAGCTTGCATCACCTTCT GCATCGCTTCTACCGCTGTCGTCGCTGTCAA CGCTTCTTACTACCACCATCACCACCATCAC GACTACGACTACCCTTACGACGTGCCTGACT ACGCTATCCCTACCACCGAAAACCTGTACTT CCAGTCTGGTACCGCGTTCTAAATGTACCCG TCAGGATGCTAGCTAA |

## Supplementary Table 2. Diffraction data processing summary.

| **Dataset** | **HEX-1 cyto** | **HEX-1 cyto** | **HEX-1 ori** | **HEX-1 ori** | **HEX-1 cyto v2** | **IMPDH ori** | **IMPDH ori** | **IMPDH cyto** |
|---|---|---|---|---|---|---|---|---|
| Data collection date | 2017/07/02 | 2021/08/24 | 2019/11/26 | 2019/11/26 | 2021/08/24 | 2019/11/26 | 2019/11/26 | 2019/09/17 |
| Sample temperature [K] | 296 | 100 | 100 | 100 | 100 | 100 | 100 | 100 |
| Processing software | CrystFEL | CrystFEL | CrystFEL | XDS | CrystFEL | CrystFEL | XDS | CrystFEL |
| PDB code | 8C5K | 8CD4 | 8CD5 | 8CGX | 8CD6 | 8C53 | 8CGY | 8C51 |
| Space group | P65 2 2 | P65 2 2 | P65 2 2 | P65 2 2 | P65 2 2 | P4 21 2 | P4 21 2 | P4 21 2 |
| Unit cell | 58.65 58.65 191.46 90 90 120 | 57.39 57.39 189.96 90 90 120 | 57.23 57.23 198.15 90 90 120 | 57.24 57.24 198.19 90 90 120 | 56.7 56.7 196.8 90 90 120 | 207.12 207.12 92.54 90 90 90 | 207.1 207.1 92.5 90 90 90 | 205.77 205.77 92.08 90 90 90 |
| Resolution range | 49.09 - 2.16 (2.237 - 2.16) | 50.19 - 1.83 (1.895 - 1.83) | 48.08 - 1.56 (1.616 - 1.56) | 33.03 - 1.85 (1.916 - 1.85) | 47.64 - 1.85 (1.916 - 1.85) | 57.38 - 2.30 (2.33 - 2.299) | 92.62 - 3.0 (3.107 - 3.0) | 77.82 - 2.40 (2.456 - 2.399) |
| Total reflections | 2,954,673 | 24,754,589 | 210,384,917 | 80,449,866 | 4,735,984 | 326,687,476 | 97,508,239 | 139,358,146 |
| Unique reflections | 11,210 (1,066) | 17.908 | 28.553 | 17,305 (1,674) | 17 | 89,070 (5,857) | 40,798 (9,383) | 77,541 (5,119) |
| Multiplicity | 246 (68) | 1,382 (100) | 7,367 (610) | 4,639 (4,197) | 278 (115.9) | 3,667 (1,246) | 474 (2,177) | 1,797 (1,018) |
| Completeness (%) | 99.96 (99.72) | 99.92 (99.31) | 99.83 (98.82) | 99.8 (98.3) | 99.81 (99.09) | 99.88 (99.09) | 99.8 (99.2) | 99.92 (99.32) |
| SNR/ < I/σ(I)> | 10.41 (0.90) | 22.16 (0.64) | 31.47 (0.59) | 35.15 (3.30) | 8.15 (0.53) | 10.06 (0.58) | 20.37 (4.21) | 9.13 (0.89) |
| Wilson B-factor | 24,10 | 31,70 | 27,56 | 31,60 | 34,14 | 53.06 | 53.76 | 61,37 |
| R-meas | - | - | - | 117.0 (56,232.3) | - | - | 341.1 (8,831.4) | - |
| R-split | 5.86 (87.1) | 3.40 (168.07) | 1.95 (188.97) | - | 7.95 (184.98) | 8.62 (199.52) | - | 8.90 (180.99) |
| CC1/2 | 0.998 (0.595) | 0.9994 (0.2745) | 0.9997 (0.2865) | 0.999 (0.939) | 0.9953 (0.4226) | 0.9976 (0.1957) | 0.981 (0.870) | 0.9973 (0.2331) |
| CC* | 0.999 (0.864) | 0.9998(0.6564) | 0.9999 (0.6674) | - | 0.9988 (0.7707) | 0.9994 (0.5722) | - | 0.9993 (0.6149) |
| **Refinement** | | | | | | | | |
| No. collected images | 595,419 | 119,053 | 56,891 | 56,891 | 114,498 | 96,949 | 96,949 | 82,534 |
| Hits / lattices | 91,354/ 57,052 | 44,200/41,591 | 54,077/131,442 | 7,922 ind. crystals (each up to 7 cons. frames) after filtering | 24,520/21,835 | 75,741/82,831 | 6,001 ind. crystals (each up to 11 cons. frames) after filtering | 55,854/71,502 |
| Lattices after stream_grep | 38,394 | - | - | - | 6,117 | - | - | - |

| | | | | | | | | |
|---|---|---|---|---|---|---|---|---|
| Removed salt intensities | - | 0.02 % | 0.03 % | - | 0.01 % | 7.6 % | - | 3.8 % |
| Reflections used in refinement | 11,207 (1,066) | 17,807 (1,718) | 28,419 (2,729) | 18,624 (1,699) | 16,899 (1,631) | 88932 (8710) | 40,788 (3982) | 73698 (7591) |
| Reflections used for R-free | 1,120 (106) | 1,323 (127) | 1,473 (139) | 1,268 (117) | 1,045 (102) | 1078 (107) | 1,598 (156) | 1058 (109) |
| R-work | 0.182 (0.320) | 0.1923 (0.3548) | 0.1993 (0.4334) | 0.2061 (0.3500) | 0.2034 (0.3964) | 0.2132 (0.3772) | 0.2014 (0.3415) | 0.2097 (0.3853) |
| R-free | 0.231 (0.362) | 0.2148 (0.3489) | 0.2159 (0.4060) | 0.2352 (0.4580) | 0.2369 (0.4258) | 0.2431 (0.3386) | 0.2388 (0.4156) | 0.2372 (0.4257) |
| Number of non-hydrogen atoms | 1.123 | 1.185 | 1.226 | 1202 | 1.174 | 7.081 | 6.852 | 6.887 |
| Macromolecules | 1,100 | 1.095 | 1.128 | 1.124 | 1.090 | 6.799 | 6.716 | 6.791 |
| Ligands | 0 | 0 | 0 | 0 | 0 | 182 | 182 | 182 |
| Solvent | 23 | 90 | 98 | 78 | 84 | 148 | 2 | 72 |
| Protein residues | 141 | 143 | 147 | 148 | 143 | 894 | 894 | 896 |
| RMS bonds (Å) | 0,016 | 0,011 | 0,004 | 0,006 | 0,008 | 0,004 | 0,005 | 0,005 |
| RMS angles (°) | 1.26 | 1,02 | 0,71 | 0,86 | 0,91 | 0,62 | 0,63 | 0,67 |
| Ramachandran favoured (%) | 96.4 | 97,87 | 98,62 | 97,95 | 98,58 | 97,4 | 95,46 | 96,04 |
| Ramachandran allowed (%) | 44715 | 2,13 | 0,69 | 1,37 | 1,42 | 2,6 | 4,54 | 3,85 |
| Ramachandran outliers (%) | 0 | 0 | 0,69 | 0,68 | 0 | 0 | 0 | 0,11 |
| Rotamer outliers (%) | 0 | 0 | 0 | 0 | 0 | 0,97 | 0,14 | 0,83 |
| Clash score | 3.15 | 3,65 | 4,44 | 4,03 | 6,89 | 5,26 | 12,78 | 6,15 |
| Average B-factor | 57.79 | 39,19 | 41,25 | 42,06 | 40,88 | 59,98 | 52,12 | 68,55 |
| Macromolecules | 57.94 | 38,64 | 40,6 | 41,65 | 40,27 | 59,87 | 51,77 | 68,29 |
| Ligands | - | - | - | - | - | 61,64 | 58,01 | 75,04 |
| Solvent | 50.71 | 45,94 | 48,67 | 47,91 | 48,73 | 57,22 | 34,41 | 64,03 |
| TLS refinement groups | - | 2 | 8 | 3 | - | - | 11 | 12 |
| Mean RMSD to ref. structure (6rfu f. IMPDH; 7asx f. HEX-1) | 0,4990 | 0,2600 | 0,4240 | 0,4226 | 0,5203 | 0,3650 | 0,4143 | 0,4150 |
| Max RMSD to ref. structure (6rfu f. IMPDH; 7asx f. HEX-1) | 2,7690 | 0,6520 | 2,1740 | 2,1700 | 2,0840 | 7,0810 | 7,0310 | 7,0040 |

**Supplementary Table 3. Overview of all cloning primers used.**

| Name | Sequence (5' → 3') |
|---|---|
| **CatB fwd** | GATCGGATCCATGCATCTCATGCGTGCCT |
| **CatB KDEL rev** | GATCCTCGAGCTACAGCTCATCCTTCGCCGTGTTGGG |
| **IMPDH v1 fwd** | GAAAACACCAACCTACGCACCA |
| **IMPDH v1 rev** | GGCAAAGAGTTTCCTCTCGTAGTGG |
| **IMPDH QC fwd** | GCGCTGGCGGTTGGAGCCAACGTGGCGATG |
| **IMPDH QC rev** | CATCGCCACGTTGGCTCCAACCGCCAGCGC |
| **EGFP-μNS v2 fwd** | GATCGGTACCATGGTGAGCAAGGGC |
| **EGFP-μNS v2 rev** | GATCGCTAGCCAATCGTACGTTAGCGGAACG |
| **HA C-term check rev** | CATCTTTGGCGTAGTCGGG |
| **HA C-term cyto fwd** | GATCGGATCCATGGGCGCCTACCCCTACGACGTGCCCGA |
| **HA C-term cyto rev** | GATCAAGCTTTTAAGCGTAGTCGGGCACGTCGTAGGGGT |
| **HA C-term fwd** | GCCTACCCCTACGACGTGCCCGACTAC |
| **HA C-term rev** | GTAGTCGGGCACGTCGTAGGGGTAGGC |
| **HA N-term check rev** | CTTTGGCGCCAGCGTAG |
| **HA N-term cyto fwd** | GATCGGATCCATGTACCCCTACGACGTGCCCGACTACGC |
| **HA N-term cyto rev** | GATCAAGCTTTTAGGCGCCAGCGTAGTCGGGCACGTCGT |
| **HA N-term fwd** | TACCCCTACGACGTGCCCGACTACGCTGGC |
| **HA N-term rev** | GCCAGCGTAGTCGGGCACGTCGTAGGGGTA |
| **pFB1 fwd Seq v1** | TAAAATGATAACCATCTCGC |
| **pFB1 fwd Seq v2** | TTCATACCGTCCCACCATCG |
| **pFB1 fwd Seq v3** | GTTGGCTACGTATACTCCGGA |
| **pFB1 rev Seq** | TTCAGGTTCAGGGGGAGGTG |
| **pFB1 rev Seq v2** | ACAAACCACAACTAGAATGCAGTG |
| **luciferase v1 fwd** | GAAGACGCCAAAAACATAAAGAA |
| **luciferase$^-$ v1 rev** | CTTTCCGCCCTTCTTGGC |
| **luciferase$^+$ v1 rev** | CAATTTGGACTTTCCGCCCTTC |
| **luciferase QC fwd** | GAAAGGCCCGGCTCCATTCTATCCTC |
| **luciferase QC rev** | GAGGATAGAATGGAGCCGGGCCTTTC |
| **HEX-1 v1 fwd** | TACTACGACGACGACGCTCACG |
| **HEX-1 ori fwd** | CTAGGGATCCATGGGCTACTACGACGACGAC |
| **HEX-1 ori rev** | CTAGAAGCTTTTAGAGGCGGGAACCGTGG |
| **HEX-1 v1 rev** | GAGGCGGGAACCGTGGACG |
| **HEX-1 v2 fwd** | CTAGGGTACCGGCTACTACGACGACGACG |
| **HEX-1 v2 rev** | CTAGGCTAGCGAGGCGGGAACCGTGG |
| **pUC/M13 fwd** | CCCAGTCACGACGTTGTAAAACG |
| **pUC/M13 rev** | AGCGGATAACAATTTCACACAGG |
| **FP v1 fwd** | GTGAGCAAGGGCGAGGAG |
| **FP v1 rev** | CTTGTACAGCTCGTCCATGCCG |
| **mTurq v2 fwd** | GATCGCTAGCGTGAGCAAGGGCGAG |
| **mTurq v2 rev** | GATCAAGCTTACTTGTACAGCTCGTCC |

**Supplementary Table 4. Diffraction data collection settings.** Parameters of *in cellulo* SSX at EMBL beamline P14 (PETRAIII, DESY, Hamburg) using an EIGER X detector, including crystal sizes determined by LM.

| | **HEX-1 ori** | **HEX-1 cyto** | **HEX-1 cyto** | **HEX-1 cyto v2** | **IMPDH ori** | **IMPDH cyto** |
|---|---|---|---|---|---|---|
| date | 2019/11/26 | 2021/08/24 | 2017/07/02 | 2021/08/24 | 2019/11/26 | 2019/09/17 |
| sample temp [K] | 100 | 100 | 296 | 100 | 100 | 100 |
| X-ray focus (FWHM) [µm] | 10 x 5 | 7 x 3 | 10 x 5 | 7 x 3 | 10 x 5 | 10 x 5 |
| photon flux [ph/s] | $1.6 \times 10^{13}$ | $1.6 \times 10^{13}$ | $1.1 \times 10^{13}$ | $1.6 \times 10^{13}$ | $1.7 \times 10^{13}$ | $1.5 \times 10^{13}$ |
| photon energy [keV] | 12.7 | 12.7 | 12.7 | 12.7 | 12.7 | 12.65 |
| exposure time [ms] | 100 | 20 | 1.4 | 20 | 50 | 100 |
| rotation [°/frame] | 1 | 1 | 0.05 | 1 | 1 | 0.5 |
| Crystal size [µm] | 9.7 x 7.9 | 26.3 +/- 10.2 x 5.3 +/- 1.5 | 26.3 +/- 10.2 x 5.3 +/- 1.5 | 18.5 x 5.6 | 48.0 +/- 17.0 x 6.3 +/- 1.8 | 59.7 +/- 21.1 x 4.4 +/- 1.6 |
| grid spacing [µm] | 10 x 3 | 7 x 3 | 10 x 5 | 7 x 3 | 10 x 3 | 10 x 2 |
| detector mode | 16M | 16M | 4M | 16M | 16M | 16M |
| detector distance / cutoff | 253.07 mm / 1.8 Å | 236.06 mm / 1.7 Å | 184.34 mm / 2.483 Å + 185.78 mm / 2.5 Å | 236.06 mm / 1.7 Å | 288.43 mm / 2.0 Å | 372.8 mm / 2.5 Å |

**Supplementary Table 5:** Parameters used for peak detection in the CrystFEL suite.

| | **HEX-1 ori cryo** | **HEX-1 cyto cryo** | **HEX-1 cyto RT** | **HEX-1 cyto v2 cryo** | **IMPDH ori cryo** | **IMPDH cyto cryo** |
|---|---|---|---|---|---|---|
| threshold | 0 | 0 | 61 | 0 | 75 | 81 |
| min-snr | 3 | 3.5 | 4.5 | 3 | 3.1 | 3.5 |
| min-pix | 4 | 3 | 2 | 4 | 2 | 2 |
| max-pix | 50 | 40 | 11 | 50 | 25 | 23 |
| min-res | 50 | 50 | 3 | 50 | 50 | 50 |
| max-res | 2000 | 2000 | 1600 | 2000 | 1600 | 1600 |
| min. peaks | 20 | 20 | 5 | 20 | 9 | 20 |
| int-radius | 3/5/6 | 3/5/7 | 3/4/5 | 3/5/7 | 3.6/5/7 | 3.6/5/6 |
