# Supplementary material for: A streamlined approach to structure elucidation using in cellulo crystallized recombinant proteins, InCellCryst
Source: Nat Commun. 2024 Feb 24;15:1709. doi: 10.1038/s41467-024-45985-7 (PMC10894269; doi:10.1038/s41467-024-45985-7)
Supplement: Supplementary file 3 — Description of Additional Supplementary Files [file 41467_2024_45985_MOESM3_ESM.pdf]

Description of Additional Supplementary Materials

File Name: Supplementary Data 1

Description: Calculation of viral titer (TCID50/mL) (Reed-Muench method)

File Name: Supplementary Data 2

Description: XDS-script to identify crystal wedges

File Name: Supplementary Data 3

Description: XDS-script to check for overlapping crystals
