# Supplementary material for: A streamlined approach to structure elucidation using in cellulo crystallized recombinant proteins, InCellCryst
Source: Nat Commun. 2024 Feb 24;15:1709. doi: 10.1038/s41467-024-45985-7 (PMC10894269; doi:10.1038/s41467-024-45985-7)
Supplement: Supplementary file 7 — Reporting Summary [file 41467_2024_45985_MOESM7_ESM.pdf]

Corresponding author(s): Lars Redecke
Last updated by author(s): Dec 20, 2023

Reporting Summary

Nature Portfolio wishes to improve the reproducibility of the work that we publish. This form provides structure for consistency and transparency in reporting. For further information on Nature Portfolio policies, see our Editorial Policies and the Editorial Policy Checklist.

Statistics

For all statistical analyses, confirm that the following items are present in the figure legend, table legend, main text, or Methods section.

n/a	Confirmed	
☐	☒	The exact sample size (*n*) for each experimental group/condition, given as a discrete number and unit of measurement
☐	☒	A statement on whether measurements were taken from distinct samples or whether the same sample was measured repeatedly
☒	☐	The statistical test(s) used AND whether they are one- or two-sided<br>*Only common tests should be described solely by name; describe more complex techniques in the Methods section.*
☒	☐	A description of all covariates tested
☒	☐	A description of any assumptions or corrections, such as tests of normality and adjustment for multiple comparisons
☐	☒	A full description of the statistical parameters including central tendency (e.g. means) or other basic estimates (e.g. regression coefficient) AND variation (e.g. standard deviation) or associated estimates of uncertainty (e.g. confidence intervals)
☒	☐	For null hypothesis testing, the test statistic (e.g. *F*, *t*, *r*) with confidence intervals, effect sizes, degrees of freedom and *P* value noted<br>*Give P values as exact values whenever suitable.*
☒	☐	For Bayesian analysis, information on the choice of priors and Markov chain Monte Carlo settings
☒	☐	For hierarchical and complex designs, identification of the appropriate level for tests and full reporting of outcomes
☒	☐	Estimates of effect sizes (e.g. Cohen's *d*, Pearson's *r*), indicating how they were calculated

Our web collection on statistics for biologists contains articles on many of the points above.

Software and code

Policy information about availability of computer code

Data collection	mxCuBE v2 user interface (Oscarsson et al., 2019)
Data analysis	CrystFEL software suite versions 0.9.1, 0.10.0, 0.10.1 (White et al., 2016); XDS (Coquelle et al., 2015); CsPadMaskMaker (https://github.com/kbeyerlein/CsPaPHENIX62dMaskMaker); mosflm-latt-nocell and mosflm-nolatt-cell (Powell et al., 2013); xgandalf (Gevorkov et al., 2019); TakeTwo (Ginn et al., 2016); dozor (Svensson et al., 2015); XSCALE (Kabsch 2010); Phaser (McCoy et al., 2007); PHENIX version 1.19.2-4158 (Liebschner et al., 2019); Coot version 0.9.7 (Emsley et al., 2010) adxv viewer version x86_64CentOS7, and pymol version 4.5.0. The code of the scripts developed to improve serial X-ray diffraction data processing (XDS-script to identify crystal wedges; XDS-script to check for overlapping crystals) is provided as supplemental material.

For manuscripts utilizing custom algorithms or software that are central to the research but not yet described in published literature, software must be made available to editors and reviewers. We strongly encourage code deposition in a community repository (e.g. GitHub). See the Nature Portfolio guidelines for submitting code & software for further information.

Data

Policy information about availability of data

All manuscripts must include a data availability statement. This statement should provide the following information, where applicable:

- Accession codes, unique identifiers, or web links for publicly available datasets
- A description of any restrictions on data availability
- For clinical datasets or third party data, please ensure that the statement adheres to our policy

Protein Data Bank: Coordinates and structure factors have been deposited in the RCSB Protein Data Bank (PDB, www.rcsb.org/) with accession codes PDB 8C51 (IMPDH cyto), PDB 8C53 (IMPDH ori, processed with CrystFEL), PDB 8CGY (IMPDH ori, processed with XDS), PDB 8CD5 (HEX-1 ori, processed with CrystFEL), PDB 8CGX (HEX-1 ori, processed with XDS), PDB 8CD4 (HEX-1 cyto, 100 K), PDB 8C5K (HEX-1 cyto, RT), PDB 8CD6 (HEX-1 cyto v2). We have referred to the previously published PDB codes 7ASX, 1JCN, 6RFU, and 1KHI.

Research involving human participants, their data, or biological material

Policy information about studies with human participants or human data. See also policy information about sex, gender (identity/presentation), and sexual orientation and race, ethnicity and racism.

Reporting on sex and gender	No research involving human participants has been performed.
Reporting on race, ethnicity, or other socially relevant groupings	N/A
Population characteristics	N/A
Recruitment	N/A
Ethics oversight	N/A

Note that full information on the approval of the study protocol must also be provided in the manuscript.

Field-specific reporting

Please select the one below that is the best fit for your research. If you are not sure, read the appropriate sections before making your selection.

☒ Life sciences ☐ Behavioural & social sciences ☐ Ecological, evolutionary & environmental sciences

For a reference copy of the document with all sections, see nature.com/documents/nr-reporting-summary-flat.pdf

Life sciences study design

All studies must disclose on these points even when the disclosure is negative.

Sample size	No sample size calculation was performed. Usually, 0.5 - 2 x 10^6 identically treated cells were used for the experiments and analysed by applying light microscopy, TEM, immunofluorescence staining, X-ray powder diffraction, SAXS-XRPD, flow cytometry, and serial X-ray diffraction. The large number of individual cells was deemed sufficient to illustrate the reproducibility of the acquired results. This sample size clearly allowed to identify alterations in the cell morphology and crystal growth, as well as to record sufficiently intense X-ray diffraction data.
Data exclusions	No data was excluded from the studies
Replication	At least 3 independent experiments have been performed, which confirmed reproducibility of the data.
Randomization	Randomly selected cells within a cell culture volume were used for analysis. For light microscopy and immunofluorescence staining, all adherent cells within a fixed area of the cell culture well were allocated into an experimental group. For TEM and all X-ray diffraction experiments, all cells from a single well were allocated into an experimental group.
Blinding	Since cells within a culture volume have been identically treated, blinding was not required.

Reporting for specific materials, systems and methods

We require information from authors about some types of materials, experimental systems and methods used in many studies. Here, indicate whether each material, system or method listed is relevant to your study. If you are not sure if a list item applies to your research, read the appropriate section before selecting a response.

Materials & experimental systems

n/a	Involved in the study
☐	☒ Antibodies
☐	☒ Eukaryotic cell lines
☒	☐ Palaeontology and archaeology
☒	☐ Animals and other organisms
☒	☐ Clinical data
☒	☐ Dual use research of concern
☒	☐ Plants

Methods

n/a	Involved in the study
☒	☐ ChIP-seq
☐	☒ Flow cytometry
☒	☐ MRI-based neuroimaging

Antibodies

Antibodies used	Mouse αHA-epitope tag antibody (BioLegend 901501, clone 16B12, 1:1000 dilution); αMs DyLight 549 antibody (Jackson ImmunoResearch 115-585-003; 1:15,000 dilution)
Validation	Validation certificates were provided by Biolegend (Quality tested and validated for Western blotting, immunocytochemistry, immunoprecipitation, and flow cytometry using the Posi-Tag Control Protein (931301), Royer Y, et al. 2005. J. Biol. Chem. 29:27251; Bennett BD, et al. 2000. J Biol Chem. 275:37712; Kim Y, et al. 2016. Nat Commun. 7:10347; Passarelli MC, et al. 2022. Nat Cell Biol. 24:307), and Jackson ImmunoResearch.

Eukaryotic cell lines

Policy information about cell lines and Sex and Gender in Research

Cell line source(s)	Spodoptera frugiperda Sf9 and Trichoplusia ni High Five insect cells were purchased from Thermo Fisher Scientific Inc.
Authentication	Insect cells were not authenticated. A corresponding certificate was provided by Thermo Fisher.
Mycoplasma contamination	All insect cell lines were regularly tested for Mycoplasma contamination and the cells used in this study were confirmed to be negative.
Commonly misidentified lines (See ICLAC register)	No commonly misidentified cell lines have been used in this study.

Flow Cytometry

Plots

Confirm that:

☒ The axis labels state the marker and fluorochrome used (e.g. CD4-FITC).

☒ The axis scales are clearly visible. Include numbers along axes only for bottom left plot of group (a 'group' is an analysis of identical markers).

☒ All plots are contour plots with outliers or pseudocolor plots.

☐ A numerical value for number of cells or percentage (with statistics) is provided.

Methodology

Sample preparation	High Five insect cells invected with recombinnat baculovirus to produce flourescent marker proteins or proteins that should crystallize within the cells were directly used from cell culture.
Instrument	LSR II (BD Bioscience) equipped with 405 nm, 488 nm, and 561 nm laser lines and Sony SH800S cell sorter equipped with 488 and 561 nm lasers.
Software	*Describe the software used to collect and analyze the flow cytometry data. For custom code that has been deposited into a community repository, provide accession details.*
Cell population abundance	*Describe the abundance of the relevant cell populations within post-sort fractions, providing details on the purity of the samples and how it was determined.*
Gating strategy	Fluorescent or crystal contianing cells were sorted based on forward and side scatter analysis or on fluorescence intensity. No further gating strategies have been applied.

☐ Tick this box to confirm that a figure exemplifying the gating strategy is provided in the Supplementary Information.
